# Detection of Somatic Mutations in Exome Sequencing of Tumor-only Samples

**DOI:** 10.1038/s41598-017-14896-7

**Published:** 2017-11-21

**Authors:** Yu-Chin Hsu, Yu-Ting Hsiao, Tzu-Yuan Kao, Jan-Gowth Chang, Grace S. Shieh

**Affiliations:** 1 Institute of Statistical Science, Academia Sinica, Taipei 115, Taiwan, R.O.C.; 2 Department of Laboratory Medicine, China Medical University and Epigenome Research Center, China Medical University Hospital, Tai-Chung 404, Taiwan, R.O.C.

## Abstract

Due to lack of normal samples in clinical diagnosis and to reduce costs, detection of small-scale mutations from tumor-only samples is required but remains relatively unexplored. We developed an algorithm (GATKcan) augmenting GATK with two statistics and machine learning to detect mutations in cancer. The averaged performance of GATKcan in ten experiments outperformed GATK in detecting mutations of randomly sampled 231 from 241 TCGA endometrial tumors (EC). In external validations, GATKcan outperformed GATK in TCGA breast cancer (BC), ovarian cancer (OC) and melanoma tumors, in terms of Matthews correlation coefficient (MCC) and precision, where MCC takes both sensitivity and specificity into account. Further, GATKcan reduced high fractions of false positives detected by GATK. In mutation detection of somatic variants, classified commonly by VarScan 2 and MuTect from the called variants in BC, OC and melanoma, ranked by adjusted MCC (adjusted precision) GATKcan was the top 1, followed by MuTect, VarScan 2 and GATK. Importantly, GATKcan enables detection of mutations when alternate alleles exist in normal samples. These results suggest that GATKcan trained by a cancer is able to detect mutations in future patients with the same type of cancer and is likely applicable to other cancers with similar mutations.

## Introduction

Advances in both next generation sequencing (NGS) technologies and computational tools have transformed biological and medical research over the past few years. In particular, calling somatic mutations from DNA sequencing data of tumor samples has become essential for characterizing cancer genomes and clinical genome typing^1,2^. Exome-sequencing (exome-seq) has enabled rapid detection of mutations that altered protein functions across hundreds of patients. However, identifying small-scale mutations consisting of somatic single nucleotide variations (SNVs) and insertions and deletions (indels) of exome-seq data is challenging, because sequencing coverage is non-uniform across target regions and among samples, the genomes of primary tumors are genetically heterogeneous, and so on^3^. Several algorithms have been developed to tackle these challenges, and they can be classified into two groups: (1) calling variants in tumor and normal samples separately, then identifying tumor-specific variants by a simple subtraction method, e.g., GATK^4^; and (2) analyzing tumor-normal samples simultaneously by heuristic methods or statistical models, e.g., Strelka^5^, VarScan 2^3^ and MuTect^6^. The algorithms in the second category can detect small-scale mutations with enhanced accuracy. In particular, MuTect focuses on detecting low-allele-frequency somatic mutations, which are often missed by existing methods, in exome-seq data requiring only a few supporting reads. VarScan 2 outperformed MuTect and other tools for variants with allele frequency >0.35, while MuTect outperformed the other five algorithms for identifying mutations with allele frequency $$\le $$ 0.35, as shown by simulated data^7^. The algorithms in the second category can be applied only when tumor-normal paired samples are available. However, most of the exome-seq data from clinical diagnosis and formalin-fixed, paraffin-embedded samples are tumor-only. As artifacts of called variants generated either from next-generation sequencing machines (accuracy limited to one error in 100 or 1000 bases) or from variant-calling algorithms remain inevitable, and validation of variants is costly (US$5–10 per variant in Taiwan), developing an algorithm to accurately detect somatic mutations of exome-seq from tumor-only samples is of interest. Moreover, detecting mutations with high accuracy may provide clues to identify driver genes in cancer^8–10^, which may reveal the mechanism of carcinogenesis.

GATK is good at discovering all potential variants across diverse sequencing technologies and experimental designs. GATK trained by known polymorphic sites performs well in capturing true single nucleotide polymorphisms (SNPs), but may produce false positives in detecting somatic mutations in exome-seq of tumor-only samples (a pilot study of endometrial tumors in Taiwan; unpublished data). Here, we developed an algorithm based on GATK^4,11^ and partial reported mutations of endometrial cancer (EC) in The Cancer Genome Atlas (TCGA)^2^, and named it GATK for cancer (GATKcan). Specifically, we incorporated two statistics to filter false mutations and detect true mutations from called variants, in addition to four statistics in hard filtering of GATK. Next, we trained the thresholds of the six statistics using partial randomly sampled TCGA endometrial tumors and machine learning. To evaluate the stability of GATKcan’s performance, we repeated the training procedure ten times and compared the averaged performance of GATKcan in detecting mutations of the remaining 231 TCGA endometrial tumors to that of GATK. We further compare GATKcan to GATK, VarScan 2 and MuTect in predicting somatic variants, classified commonly by VarScan 2 and MuTect, from the called variants. Moreover, the four algorithms were compared using exome-seq data of 215 ovarian tumors, 503 breast cancer tumors and 342 samples in melanoma of TCGA^12–14^. Detecting small-scale mutations when alternative alleles in normal samples exist has been a bottleneck in the area. Because GATKcan does not require normal samples, this problem is circumvented using our approach.

## Results

For this study, we incorporated exome-seq of EC tumors (~95% non-Asian) from TCGA^2^, which was part of 373 endometrial carcinomas consisting of genomic, transcriptomic and proteomic profiling^2^. This integrated characterization provided key molecular insights into tumor classification. We first applied HaplotypeCaller to yield variant calls, that HaplotypeCaller compared with a reference genome (hg19) to sift variants. Specifically, a total of 64,295 variants (base quality $\ge$ 10 and MQ $\ge$ 20) were called by GATK from exome-seq of 241 samples (focusing on ~800 cancer genes (~1GB per sample)); seven of the 248 files were damaged after downloading. The list of cancer genes studied is shown in Supplementary Table S1. Of these called variants, 64,183 were classified as point mutations and 112 were indels by GATK. Calling variants of each sample took GATK ~2 h using a multi-core cluster (2 Xeon 2.67 GHz CPUs and 24GB RAM). The details of the datasets are shown in Table 1.

**Table 1:** Exome-seq datasets summary.

	EC (Exome-seq)	OC (Exome-seq, WUGSC)	OC (Exome-seq, BI)
No. of samples	248	79	136
Sequencing technology	Illumina GAIIx or Hiseq. 2000	Illumina GAIIx or ABI 3730	Illumina GAIIx
Coverage per sample	at least 20x	at least 20x	at least 20x
Read architecture	100 bp paired end	100 bp paired end	76 bp paired end
Target area	whole exome	whole exome	whole exome
Data set source	TCGA Research Network	TCGA Research Network	TCGA Research Network
Aligner	BWA	BWA	Picard
	**BC (Exome-seq)**	**Cutaneous Melanoma (Exome-seq)**	
No. of samples	503	342	
Sequencing technology	Illumina Hiseq. 2000	Illumina HiSeq. 2000	
Coverage per sample	~20x	~82x	
Read architecture	100 bp paired end	76 bp paired end	
Target area	whole exome	whole exome	
Data set source	TCGA Research Network	TCGA Research Network	
Aligner	BWA	BWA/Picard	

GATK is very good at uncovering potential variants and filtered machine artifacts of DNA sequencing data. HaplotypeCaller of GATK is very useful for calling single nucleotide polymorphisms (SNPs) and indels of DNA sequencing data from diseased-only samples and paired samples. After applying HaplotypeCaller to call variants, we excluded known SNPs in the HapMap3 and the 1000 Genomes Project^15^ to result in potential somatic variants in tumors. Note that we did not use dbSNP, because it contained some verified somatic mutations which were of interest to us. We then inputted these variants to hard filtering of GATK, using the following five statistics to identify somatic mutations; HaplotypeScore was excluded because it had been taken into account during the calling process.

- QualByDepth (QD): this is the quality of the variant divided by the unfiltered depth of non-reference samples.
- FisherStrand (FS): Phred-scaled P value of Fisher’s exact test to detect strand bias (the variant being seen on only the forward or only the reverse strand) in the reads.
- RMSMappingQuality (MQ): this is the root mean square of the mapping quality of the reads across all samples.
- MappingQualityRankSumTest (MQRankSum): this is the z-approximation from the Mann-Whitney rank sum test for mapping qualities on reads with reference (REF) bases versus those with alternate (ALT) alleles.
- ReadPosRankSum Test (ReadPosRankSum): the z-approximation from the Mann-Whitney rank sum test^16^ for the distance from the end of the read for reads with the alternate allele. If the alternate allele is only seen near the ends of reads, this is indicative of error.

Hard filtering classifies a called variant with QD < 2.0, FS > 60.0, MQ < 40.0, MQRankSum < −12.5 or ReadPosRankSum < −8.0 (QD < 2.0, FS > 200.0 and ReadPosRankSum < −20.0) to be an artifact, and otherwise to be a SNP (an indel). To see whether all five statistics were useful for identifying somatic mutations, we conducted a pilot study by applying hard filtering with the aforementioned default thresholds^4,11^ to 241 EC tumors from TCGA. The artifacts filtered by ReadPosRankSum were a subset of those filtered by FS, so we only incorporated QD, FS, MQ and MQRankSum into our method.

### The proposed method—GATK for cancer (GATKcan)

Because validation of somatic mutation is costly, we introduced two more statistics to filter false mutations and identify true mutations from called variants, in addition to the above four statistics of hard filtering. Further, we were able to assess Level 1 exome-seq data of 241 endometrial tumors from TCGA, thus we trained the thresholds of the six statistics using known mutations of partial TCGA EC, reported mutations in 19 TCGA cancer types and applied GATKcan to detect somatic mutations of the remaining EC tumors. Further, we also applied GATKcan to detect mutations in similar carcinoma (ovarian cancer and breast cancer) and a tumor of a different carcinoma (melanoma; squamous cell).

For a called variant, if the differences in the number of reads from 5′ and 3′ deviate from those of its corresponding REF a lot, then it is likely to be a false mutation. We assume that mutation sites for some tumors of a cancer are the same. For each called variant, we incorporated the Mann-Whitney statistic to test whether its differences in the number of reads from both strains have the same distribution as those of the corresponding REF across tumors; ideally the latter have median zero. If the hypothesis is rejected, then we predict this variant as a false mutation. Moreover, somatic mutation rate is rare (~2.8 × 10^−7^ per base^17^), thus the probability of an adjacent mutation existing within the neighborhood of a true mutation is very small. The intermutation distance (IMD) is defined as the distance from one mutation to the next one^18^. The IMDs calculated from cancer genes of 248 EC, 510 BC, 316 OC and 346 melanoma tumors are huge, and their median are ~17.5, 25.3, 2.7 and 3.4 Mb, respectively. Therefore, for a given variant if the genomic distance from its nearest reported mutation is small but >0 (=0), then we can classify it as a false (true) mutation. We also incorporated this distance (called dNM) and determined its threshold by a machine learning approach. Therefore, in addition to QD, FS, MQ and MQRankSum (QD and FS) for detecting point mutations (indels), we incorporated the following two statistics into our algorithm GATKcan.

1. Mann-Whitney test, and
2. dNM: a genomic distance of a called variant from its nearest true mutation.

Note that when a dNM equaled to zero, namely this variant coincided with a reported mutation in 19 TCGA cancer types, we predicted it as a true mutation and did not filter it further. Note that dNM and these trained thresholds enabled GATKcan to identify true mutations, in addition to filter false mutations.

Because this study was originally motivated by an analysis of ten EC tumors in Taiwan (results not shown), when we gained access to exome-seq data of TCGA endometrial tumors, it was reasonable to train the thresholds of the statistics in GATKcan using these EC tumors, instead of using the fixed cutoffs of hard filtering. To compare with training using Taiwanese ECs and to reduce training time, we used (~2788 called variants of) ten randomly selected TCGA EC tumors for training. Specifically, we trained the thresholds of the six statistics of GATKcan using the reported mutations and false mutations (false positives) of the called variants in randomly sampled 2, 3, 2 and 3 EC tumors of stage I-IV, respectively (namely adopting a stratified sampling scheme). The 64,295 called variants did not contain any reported indel, thus we used ~9% of 539 reported indels (from all reported mutations) and 112 false mutations (from the called variants) to train the four cutoffs of GATKcan for detection of indels; see Methods for further details of the training procedure. To assess stability of GATKcan’s performances, we repeated the training procedure ten times and obtained ten sets of trained thresholds in Table 2.

**Table 2:** (A) The six thresholds of GATKcan trained by randomly sampled 10 TCGA EC tumors and performances of GATKcan in the ten training experiments. (B) The four thresholds of GATKcan trained by ~10% of 539 reported indels and 112 artifacts (from 241 TCGA EC tumors), and performances of GATKcan in the ten training experiments.

Repeat	α	dNM	FS	MQ	MQRankSum	QD	Mann-Whitney test (P value)	Training	
								TPR	cFPR
**A. The thresholds of GATKcan for detection of single nucleotide variations**									
1	0.5	991.1	46.1	50.0	−7.85	0.11	0.010	98.6	10.7
2	0.5	988.1	43.6	49.5	−7.90	0.12	0.010	98.1	10.7
3	0.5	990.5	45.8	39.2	−10.10	0.05	0.010	99.3	14.3
4	0.3	985.6	49.3	48.2	−10.14	0.08	0.010	98.6	12.2
5	0.3	984.3	51.8	48.0	−10.37	0.09	0.009	98.9	11.6
6	0.4	981.5	50.9	50.0	−9.59	0.09	0.010	98.7	10.8
7	0.4	989.3	45.7	39.9	−8.97	0.05	0.082	99.3	14.0
8	0.3	982.3	55.2	50.0	−8.36	0.18	0.010	99.6	11.9
9	0.5	987.9	47.0	49.9	−10.22	0.11	0.010	98.2	10.5
10	0.5	989.1	45.6	39.9	−9.58	0.04	0.010	99.2	14.3
**B. The thresholds of GATKcan for detection of indels**									
**Repeat**	**α**	**dNM**	**FS**	**QD**	**Mann-Whitney test (P value)**	**Training**			
						**TPR**	**cFPR**		
1	0.5	753.4	86.3	0.3	0.055	100.0	0.0		
2	0.5	405.0	133.3	0.4	0.090	100.0	0.0		
3	0.3	581.6	91.6	0.3	0.055	100.0	0.0		
4	0.5	395.2	136.6	0.8	0.064	100.0	0.0		
5	0.5	658.8	102.7	0.4	0.013	100.0	0.0		
6	0.6	464.0	121.0	0.2	0.037	100.0	0.0		
7	0.3	397.6	95.1	0.2	0.027	100.0	0.0		
8	0.3	619.2	99.1	0.4	0.046	100.0	0.0		
9	0.5	566.9	135.7	0.5	0.100	100.0	0.0		
10	0.5	633.6	139.9	0.8	0.003	100.0	0.0		

For each cancer under study, we focused on a few hundreds of cancer genes, whose DNAs consisted of reference (non-mutated) sites and called variants. Excluding the reported SNPs and germline mutations in ExAC^19^, we defined a true mutation as a true positive (TP) and a false mutation in called variants as a true negative (TN). The true positive rate (TPR, namely sensitivity) is defined as the ratio of the identified TPs to the total number of TPs (reported by TCGA). Similarly, precision is the ratio of the identified TPs to all predicted mutations, false positive rate (FPR, namely $1-$ specificity) is the ratio of the predicted mutations to all REF sites of cancer genes in all tumors under test, and conditional FPR (cFPR) is the ratio of predicted mutations to all TNs in the called variants. Note that cFPR is of interest in clinical diagnoses, in addition to TPR. Because there is only ~10% true mutations in the EC tumors, we further adopted Matthews correlation coefficient (MCC). MCC is a balanced measure which takes into account true and false positives (negatives), and

$${\rm{MCC}}=\frac{TPs\times TNs\,-\,FPs\times FNs}{\sqrt{(TPs+FPs)(TPs+FNs)(TNs+FPs)(TNs+FNs)}},$$

where FPs and FNs denote false positives and false negatives, respectively. Next, we compared GATKcan to hard filtering (denoted by GATK henceforth) in detection of mutations of the remaining 231 tumors in ten repeats. The averaged TPR (cFPR) of GATK and GATKcan (trained by ten tumors) for detecting mutations of the 61,507 called variants are ~88.2% (~65.1%) and ~96.1% (~12.2%), respectively. Further, the averaged MCC (precision) of GATK and GATKcan are ~15% (~13%) and ~62% (46%), respectively. Let Mb denote megabase. The FPR of GATK and GATKcan (with the first set of cutoffs) was ~313 ${{\rm{Mb}}}^{-1}$ and ~189 ${{\rm{Mb}}}^{-1}$, respectively which were computed over randomly selected 10% of ~$6.04\times {10}^{8}$ reference sites in cancer genes of 241 EC tumors. The detailed results of the ten repeats are shown in Supplementary Table S2.

To investigate the performance of GATKcan on more training samples, we further trained GATKcan using the called variants in ~18% (44 randomly selected) of 241 TCGA EC tumors. GATKcan trained by 44 tumors performed similarly to GATKcan trained by 10 tumors, which may be due to the six statistics captures the patterns of TPs and TNs well and training by ~2788 variants (of ten samples) been sufficient; the results are shown in Table 3 and the cutoffs in Table S2.

**Table 3:** The averaged performances of GATK and GATKcan in detecting mutations from (A) ~61,507 variants of randomly sampled 231 endometrial tumors in ten repeats, and (B) ~52,291 variants of randomly sampled 197 endometrial tumors in ten repeats, checked against the 184,824 reported mutations in EC of TCGA.

		GATK		GATKcan
A.				
Training	TPR^**§**^ (s.e.)	—	-----*-----	99.0 (1.0)
	cFPR (s.e.)	—	-----*-----	11.9 (1.3)
Test	TPR (s.e.)	88.2 (0.6)	-----*-----	96.1 (1.9)
	cFPR (s.e.)	65.2 (0.1)	-----*-----	12.2 (1.6)
	precision (s.e.)	12.6 (0.4)	-----*-----	46.0 (3.2)
	MCC (s.e.)	14.5 (0.5)	-----*-----	61.8 (2.0)
B.				
Training	TPR^**§**^ (s.e.)	—	-----*-----	98.8 (0.5)
	cFPR (s.e.)	—	-----*-----	12.1 (1.6)
Test	TPR (s.e.)	88.1 (1.3)	-----*-----	96.1 (2.1)
	cFPR (s.e.)	64.9 (0.4)	-----*-----	12.3 (1.6)
	precision (s.e.)	12.6 (0.9)	-----*-----	45.6 (2.9)
	MCC (s.e.)	14.6 (1.0)	-----*-----	61.4 (1.8)

^§^The unit of all performance measures and their s.e.’s are %. *Denotes the P value of the two sample t-test < 10^−7^.

Although GATK can identify small-scale mutations reasonably well for tumor-only samples, it is known to be limited to mutations with median to high allelic fractions^4,20^, where allelic fraction was defined as the ratio of the variant reads to the total reads at a given site. Thus, it is of interest to compare GATK and GATKcan in identifying mutations of EC by allelic fractions. This may provide insights into which cases both methods can be applied adequately.

**Figure 1:**
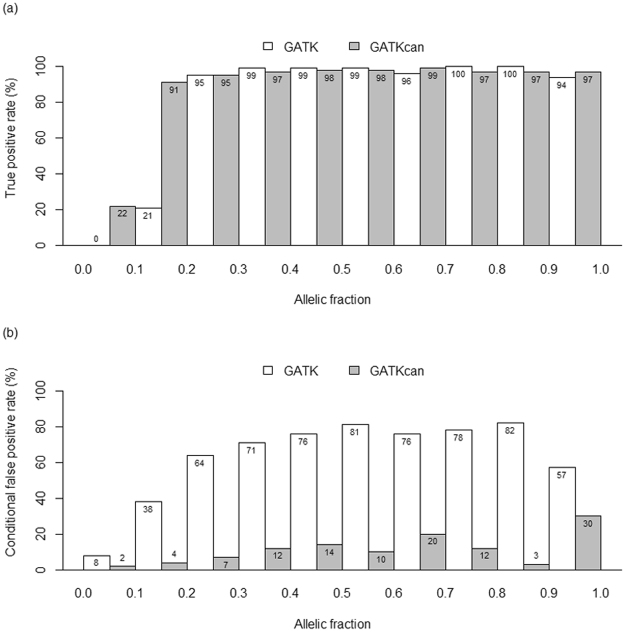
The averaged performance of GATK and GATKcan listed by allelic fractions when applied to exome-seq of 231 randomly sampled endometrial tumors in the ten repeats, where Fig. 1(a) and (b) illustrate TPR and conditional FPR of the two algorithms, respectively.

Figure 1 demonstrates that averaged over 10 repeats, GATKcan identifies true mutations well with allelic fractions $\ge$ 0.2, while GATK requires allelic fractions $\ge$ 0.3 to perform well. The TPR of GATKcan is close to those of GATK for variants with allelic fractions in (0.2, 1.0], but TPR of GATKcan is 22% and 70% higher than that of GATK for variants with allelic fraction in (0, 0.1] and (0.1, 0.2], respectively (Fig. 1a). For all variants with allelic factions in (0, 1.0], GATKcan is more powerful to detect artifacts, because of 6% to 79% lower cFPR than GATK (Fig. 1b).

Further, we compared GATK and GATKcan to VarScan 2 and MuTect; the latter two outperformed the remaining five tools in detecting small-scale mutations with allele frequency >0.35 and ≤0.35, respectively in Wang *et al*. (2013)^7^, where 10 pairs of simulated whole exome-seq samples with coverage of 100x were generated. In general, detection of low allele frequency mutations requires sufficient coverage, while exome-seq of ECs from TCGA had coverage of 20x only; therefore, it is of interest to compare the four algorithms.

In each of the 10 repeats, on average GATK and GATKcan detected mutations from 61,507 (~52,291) variants of 231 (197) tumors. Of these variants, VarScan 2 and MuTect (both default settings) classified ~2,102 (~1,741) as common somatic variants. Of these somatic variants, the high confidence mutations classified by VarScan 2 (MuTect with high-confidence mode) were treated as somatic mutations predicted by VarScan 2 (MuTect). We checked the predictions against the mutations reported by TCGA research network, and computed adjusted TPR and adjusted cFPR, which was the number of predicted true mutations (the predicted mutations) over the total true mutations (the total false mutations) of the somatic variants, in addition to adjusted precision (MCC). Of the ten experiments, the averaged adjusted TPR of GATK, GATKcan (both using tumor-only samples), VarScan 2 and MuTect were 98.8%, 98.6%, 99.6% and 94.3%, respectively, while their averaged adjusted cFPR were 81.0%, 5.7%, 60.5% and 37.9%, respectively. Ranked by adjusted MCC and adjusted precision, GATKcan was the top 1, followed by MuTect, VarScan 2 and GATK; some detailed results are shown in Table 4. The performances of the four algorithms on ~1,741 somatic variants (shown in Table 4) were similar to those on ~2,102 somatic variants; all results of the ten repeats are shown in Supplementary Table S2. On average, it took GATK and GATKcan (VarScan 2) ~6.7 h (25.8 h with 71% of jobs run in high memory cluster (2 Xeon 2.5 GHz CPUs and 384GB RAM) and 29% jobs run in a multi-core cluster) to detect mutations in 231 (paired) samples in each repeat.

**Table 4:** The averaged performances of GATK, GATKcan (trained by ten & 44 EC tumors), VarScan 2 and MuTect in detecting mutations from ~2,102 (~1,741) somatic variants of 231 (197) randomly sampled endometrial tumors in ten repeats, checked against the TCGA reported mutations.

Test set		GATK	GATKcan		Mutect	VarScan 2
231 samples	TPR^†**§**^ (s.e.)	98.8 (0.0)	98.6 (0.6)	-----*-----	94.3 (0.1)	99.6 (0.0)
	cFPR^†^ (s.e.)	81.0 (0.4)	5.7 (0.4)	-----*-----	37.9 (0.5)	60.5 (0.7)
	precision^†^ (s.e.)	54.7 (0.3)	94.4 (0.3)	-----*-----	71.1 (0.2)	61.9 (0.2)
	MCC^†^ (s.e.)	29.4 (0.4)	92.9 (0.5)	-----*-----	59.5 (0.4)	48.9 (0.5)
197 samples	TPR^†**§**^ (s.e.)	98.8 (0.3)	98.5 (1.0)	-----*-----	94.1 (1.0)	99.6 (0.1)
	cFPR^†^ (s.e.)	80.2 (2.3)	6.1 (0.9)	-----*-----	36.8 (5.2)	59.6 (4.5)
	precision^†^ (s.e.)	55.0 (0.7)	94.2 (0.6)	-----*-----	71.9 (2.1)	62.4 (0.9)
	MCC^†^ (s.e.)	30.3 (1.5)	92.5 (0.3)	-----*-----	60.3 (3.5)	49.5 (3.1)

^†^Adjusted performance measures. ^**§**^The unit of all performance measures and their s.e.’s are %. *The P value of the two sample t-test between GATKcan and MuTect < 10^−8^.

### External Validations on Three TCGA cancers

Because (1) the tumorigenesis of most cancer types are similar; (2) cancer genes bearing mutations in EC are similar to other cancer types^2,12–14^, e.g., TP53, PIK3CA and KRAS; and (3) some mutational signatures of cancers are similar^18^, we wondered how well GATKcan trained by partial TCGA EC tumors can be applied to other cancer types. Thus, we applied GATKcan, GATK, VanScan 2 and MuTect to TCGA Breast Cancer, Ovarian Cancer and Cutaneous Melanoma datasets, as three validation sets in this section. The lists of cancer genes studied are shown in Supplementary Table S1.

### Application to TCGA breast cancer data

As the first external validation, we applied GATK, GATKcan, VarScan 2 and MuTect to 507 breast cancer (BC) tumors with 429 blood derived normal and 74 normal tissues^13^; of the 507 samples, four.bam files were damaged after downloading. The whole exome-seq of 503 tumor-normal pairs (~11 GB/sample) was analyzed. For GATKcan, we used the ten sets of thresholds trained by 10 and 44 randomly sampled TCGA EC tumors, respectively. Specifically, a total of 50,799 variants (base quality $\ge$ 10 and MQ $\ge$ 20) were called by GATK from exome-seq, focusing on 488 cancer genes queried from COSMIC, of the 503 BC tumors.

The TPR (cFPR) of GATK and GATKcan (trained by ten and 44 tumors) for detecting mutations of the called variants are ~85% (~55%), ~71% (~3%) and ~71% (~3%), respectively, while the FPR of GATK and GATKcan (trained by ten tumors) are ~184 ${{\rm{Mb}}}^{-1}$ and ~94 ${{\rm{Mb}}}^{-1}$, respectively in detecting randomly selected 10% of ~$8.49\times {10}^{8}$ reference sites in the cancer genes of 503 tumors. Of the 50,799 variants, VarScan 2 and MuTect classified only 458 as common somatic variants. The adjusted TPR (adjusted cFPR) of GATK, GATKcan (trained by ten and 44 tumors), VarScan 2 and MuTect on the 458 somatic variants are ~99.3% (~68.5%), ~99.5% (~3.2%), ~99.5% (~4.7%), ~99.3% (~27%) and ~98.6% (~10.9%), respectively. Ranked by adjusted MCC and adjusted precision, GATKcan was the top 1, followed by MuTect, VarScan 2 and GATK; some detailed results are in Table 5. We note that the adjusted precision (MCC) of GATKcan (trained by 44 tumors) is slightly lower than those of GATKcan (trained by 10 tumors), which may be because the mutations in BC have different characteristics from those in EC. The results of GATKcan using ten sets of cutoffs are shown in Supplementary Table S3.

**Table 5:** The performance of GATK and GATKcan in detecting mutations of 50,799 variants from exome-seq of 503 TCGA BC tumors.

	GATK	GATKcan (s.e.)		Mutect	VarScan 2
		trained by			
		10 tumors	44 tumors		
TPR^**§**^	85.1	70.6 (0.3)	70.7 (0.2)	—	—
cFPR	55.1	3.2 (0.2)	3.4 (0.2)	—	—
precision	5.3	44.3 (1.2)	43.4 (1.6)	—	—
MCC	11.2	53.9 (0.7)	53.4 (1.0)	—	—
TPR^†**§**^	99.3	99.5 (0.3)	99.5 (0.3)	99.3	98.6
cFPR^†^	68.5	3.2 (1.0)	4.7 (0.3)	27.0	10.9
precision^†^	40.7	93.8 (1.8)	90.9 (0.5)	63.5	81.0
MCC^†^	35.0	94.9 (1.3)	92.7 (0.2)	67.5	83.9

Next, the four algorithms identified mutations from 458 somatic variants, on which TPR^†^, cFPR^†^, precision^†^ and MCC^†^ were computed. ^**§**^The unit of all performance measures and their s.e.’s are %.

### Application to TCGA ovarian cancer data

We further applied GATK, GATKcan and VarScan 2 to 215 ovarian cancer (OC) tumors after excluding 21 non-downloadable and damaged files^12^. The whole exome-seq of 215 tumor-normal pairs, whose size was ~14 GB (WUGSC) and ~27 GB (broad Institute) per sample, were analyzed. Specifically, a total of 27,167 variants (base quality $\ge$ 10 and MQ $\ge$ 20) were called by GATK from exome-seq, focusing on 432 cancer genes queried from COSMIC, of 215 OC tumors. Of the called variants, 19,182 and 7,984 (~29.4%) were point mutations and indels, respectively.

Checked against the 14,904 reported mutations of OC by TCGA, the TPR (cFPR) of GATK and GATKcan (trained by ten and 44 tumors) for detecting mutations from 27,167 called variants were about 85.2% (70.3%), 89.1% (1.1%) and 89.7% (0.7%), respectively, while the FPR of GATK and GATKcan were ~126 ${{\rm{Mb}}}^{-1}$ and ~85 ${{\rm{Mb}}}^{-1}$ in detecting randomly selected 10% of ~$2.97\times {10}^{8}$ reference sites of the cancer genes in 215 tumors, respectively. Of the called variants, VarScan 2 and MuTect classified only 178 common somatic variants, for which the adjusted TPR (adjusted cFPR) of GATK, GATKcan (trained by ten and 44 tumors), VarScan 2 and MuTect are 98.2% (86.9%), 98.2% (4.1%), 98.2% (3.9%), 100.0% (34.5%) and 100.0% (23.8%), respectively. Ranked by adjusted MCC and adjusted precision, GATKcan was the top 1, followed by MuTect, VarScan 2 and GATK; some detailed results are shown in Table 6. The results of GATKcan using ten sets of cutoffs are shown in Supplementary Table S4.

**Table 6:** The averaged performance of GATK and GATKcan in detecting mutations of 27,167 called variants (of 432 genes) from exome-seq of 215 TCGA OC tumors, checked against the reported mutations by TCGA.

	GATK	GATKcan (s.e.)		Mutect	VarScan 2
		trained by			
		10 tumors	44 tumors		
TPR^**§**^	85.2	89.1 (1.1)	89.7 (0.7)	—	—
cFPR	70.4	5.0 (0.2)	5.1 (0.3)	—	—
precision	2.9	30.5 (1.1)	30.1 (1.4)	—	—
MCC	5.0	50.4 (1.2)	50.2 (1.4)	—	—
TPR^†**§**^	98.2	98.2 (0.0)	98.2 (0.0)	100.0	100.0
cFPR^†^	86.9	4.1 (1.2)	3.9 (0.8)	34.5	23.8
precision^†^	34.2	91.7 (2.1)	92.0 (1.4)	62.5	65.9
MCC^†^	17.9	92.5 (1.7)	92.7 (1.1)	64.0	70.9

Next, the four algorithms identified mutations from 178 somatic variants, on which TPR^†^, cFPR^†^, precision^†^ and MCC^†^ were computed. ^**§**^The unit of all performance measures and their s.e.’s are %.

### Application to TCGA cutaneous melanoma data

Finally, to see whether GATKcan can be applied to a cancer with different histology from EC (adenocarcinoma), we applied GATK, GATKcan and VarScan 2 to cutaneous melanoma (squamous cancer). In total, whole exome-seq of 342 tumors with 340 blood derived normal and 2 normal tissues^14^ were analyzed (~9 GB/sample). For GATKcan, we used the 10 sets of thresholds trained by ten and 44 randomly sampled EC tumors of TCGA, respectively. Focusing on 498 cancer genes queried from COSMIC, GATK called a total of 33,053 variants (base quality $\ge$ 10 and MQ $\ge$ 20) from exome-seq of these tumor samples.

The TPR (cFPR) of GATK and GATKcan (trained by ten and 44 tumors) for detecting mutations of the called variants are about 89.9% (64.1%), 98.7% (4.4%) and 98.9% (4.6%), respectively. The FPR of GATK and GATKcan (trained by ten tumors) are ~101 ${{\rm{Mb}}}^{-1}$ and ~64 ${{\rm{Mb}}}^{-1}$, respectively in detecting randomly selected 10% of $\sim 4.69\times {10}^{8}$ reference sites in the cancer genes in 342 tumors. Of the 33,053 variants, VarScan 2 and MuTect classified 1,784 variants as common somatic variants, for which the adjusted TPR (adjusted cFPR) of GATK, GATKcan (trained by ten and 44 tumors), VarScan 2 and MuTect are about 98.5% (76.6%), 99.2% (8.8%), 99.2% (8.7%), 99.4% (57.0%) and 99.3% (47.9%), respectively. Ranked by adjusted MCC and adjusted precision, GATKcan was the top 1, followed by MuTect, VarScan 2 and GATK; some detailed results are shown in Table 7. The results of GATKcan using ten sets of cutoffs are shown in Supplementary Table S5.

**Table 7:** The averaged performance of GATK and GATKcan in detecting mutations of 33,053 variants (of 498 genes) from exome-seq of 342 TCGA melanoma tumors.

	GATK	GATKcan (s.e.)		Mutect	VarScan 2
		trained by			
		10 tumors	44 tumors		
TPR^**§**^	89.9	98.7 (0.4)	98.9 (0.3)	—	—
cFPR	64.1	4.4 (0.3)	4.6 (0.2)	—	—
precision	27.6	85.9 (0.9)	85.3 (0.6)	—	—
MCC	22.9	89.8 (0.7)	89.5 (0.5)	—	—
TPR^†**§**^	98.5	99.2 (0.3)	99.2 (0.3)	99.4	99.3
cFPR^†^	76.6	8.8 (1.2)	8.7 (1.1)	57.0	47.9
precision^†^	80.3	97.3 (0.3)	97.3 (0.3)	84.7	86.8
MCC^†^	37.3	92.4 (0.3)	92.5 (0.2)	58.4	65.3

Next, the four algorithms identified mutations from 1,784 somatic variants, on which TPR^†^, cFPR^†^, precision^†^ and MCC^†^ were computed. ^**§**^The unit of all performance measures and their s.e.’s are %.

## Discussion

Detection of mutations in exome-seq of tumor-only samples is useful for clinical diagnoses, as they can serve as a base for classifying cancer patients via molecular signatures and suggesting precision medicines. In addition to four statistics of GATK, GATKcan incorporated Mann-Whitney statistic and dNM to detect mutations, and we trained the cutoffs of GATKcan using reported mutations of ten randomly sampled TCGA endometrial tumors and reported mutations of 19 TCGA cancer types in each of ten experiments. The averaged performance of GATKcan was better than GATK in detecting mutations of the remaining 231 endometrial tumors. Further, GATKcan with such thresholds outperformed GATK in detecting mutations of 27,167 to 50,799 called variants in TCGA BC, OC and melanoma tumors in terms of MCC and precision, where MCC takes both sensitivity and specificity into account. Importantly, GATKcan reduced high fractions (about 23%, 52%, 65% and 60%) of false positives detected by GATK in the four cancers, whereas validation is costly (US$5–10 per variant in Taiwan).

Ranked by adjusted MCC and adjusted precision, GATKcan was top 1, followed by MuTect, VarScan 2 and GATK in mutation detection of somatic variants classified commonly by VarScan 2 and MuTect from the called variants of BC, OC and melanoma. Note that GATKcan does not require normal samples, thus it reduces sequencing costs to half. Further, it enables detection of mutations when alternate alleles exist in normal samples, which remains a bottleneck in the area.

To investigate the performance of GATKcan on more training samples, we further trained GATKcan using called variants in 44 random samples of 241 TCGA EC in each of ten experiments. GATKcan trained by 44 tumors performed similarly to GATKcan trained by ten tumors, which may be because GATKcan captures patterns of true positives and negatives well and ~2788 called variants in ten tumors are sufficient for training. Tables 5–7 show that GATKcan trained by EC yields better prediction results in melanoma and OC than in BC, which suggests that the mutations of EC are more similar to those in melanoma and OC than to those in BC. Thus, if we know from cancer biology that cancer A is similar to a cancer to be detected, then training GATKcan by cancer A will be effective. These results suggest that GATKcan trained by a cancer is able to detect mutations in future patients with the same type of cancer and is likely applicable to other cancers whose mutations are similar. In addition to sequence-based clinical diagnoses, GATKcan is expected to have a large number of applications such as in the Precision Medicine Initiative for Oncology recently launched in the US.

In the future, the reported mutations in all types of cancers^21^ of ICGC will be integrated with the mutations in TCGA as true mutations to check against. Then a pipeline based on GATKcan (for called variants in known mutation sites) and Variant Effect Predictor^22^ (for variants in sites with unknown mutation status^23^) will be built to detect somatic mutations of exome-seq of tumor-only samples. Finally, GATKcan may be improved further in identifying true mutations of tumors, e.g., by training GATKcan with partial reported mutations of all other cancers in TCGA and adding other statistics to capture characteristics of true mutations. We leave these topics for future research.

## Methods

### Training the cutoffs of the statistics in GATKcan

To train the six thresholds of GATKcan for detection of point mutations, the reported mutations of 241 endometrial tumors of TCGA were regarded as true mutations, and we randomly sampled 10 tumors (stratified by stage; 2, 3, 2 and 3 for stage I-IV), from which the called variants were inputted to the optimization algorithm particle swarm optimization^24^. Ten randomly sampled tumors were used for training, because CPU time was proportional to the number of variants inputted. The six cutoffs were optimized in the sense that they maximized the fitness function $\alpha$(1-cFPR) + ($1-\alpha$)TPR, with $\alpha$ varying from 0 (0.1) to 1 using 10-fold. For a given $\alpha$, the training set consisted of the true mutations and artifacts from 90% of the ten endometrial tumors, and the called variants of the remaining tumor made up the test set of the training (also called cross-validation). This step was iterated through each tumor being set as the test set, then we selected an $\alpha$ value to obtain the six thresholds for GATKcan. In each training, we used 4,000 seed points and 500 generations to run PSO, and partitioned the ranges of dNM, FS, MQ, MQRankSum, QD and P value of Mann-Whitney test into 1000, 60, 50, 100, 20 and 6 segments, respectively. In the training of dNM, we also used the reported mutations of 19 cancer types of TCGA; some details are in Supplementary Note 1. Similarly, to train the four cutoffs of GATKcan for detection of indels, we randomly sampled 50 of all 539 reported indels and 10 artifacts from the 64,295 called variants, respectively, because the called variants contained no true indels; the remaining 489 reported indels and 102 false indels constituted the test set. Then, the rest training procedure followed that of the six cutoffs of GATKcan. PSO is a well-known optimization method; for details of the method and computer complexity, please see Section 2.3.5 of Chuang *et al*. (2008)^25^. On average, each training procedure of GATKcan took ~15 h (~5 h) for the six (four) cutoffs, and was conducted by a high memory computing cluster (256GB RAM, limited to 11 cores and each with Xeon CPU 2.5 GHz).

## Electronic supplementary material

Supplementary Information
