# Supplementary material for: Detection of Somatic Mutations in Exome Sequencing of Tumor-only Samples
Source: Sci Rep. 2017 Nov 21;7:15959. doi: 10.1038/s41598-017-14896-7 (PMC5698426; doi:10.1038/s41598-017-14896-7)
Supplement: Supplementary file 1 — Supplementary Information [file 41598_2017_14896_MOESM1_ESM.pdf]

Supplementary Information

Detection of Somatic Mutations in Exome Sequencing of Tumor-only Samples

Yu-Chin Hsu[1], Yu-Ting Hsiao[1], Jan-Gowth Chang[2] and Grace S. Shieh[1,*]
[1]Institute of Statistical Science, Academia Sinica, Taipei 115, Taiwan, R.O.C.;
[2]Department of Laboratory Medicine, and Center of RNA Biology and Clinical Application, China Medical University Hospital, China Medical University, Taichung 404, Taiwan, R.O.C.

Table S1. The list of cancer genes analyzed in the four cancers

**Endometrial Cancer**	**Breast Cancer**	**Ovarian Cancer**	**Cutaneous Melanoma**
ABCC6	ABI1	ABI1	ABI1
ABI1	ABL1	ABL1	ABL1
ABL1	ABL2	ABL2	ABL2
ABL2	ACSL3	ACSL3	ACSL3
AC083949.1	ACSL6	ACSL6	ACSL6
ACKR3	ACVR1	ACVR1	ACVR1
ACSL3	AFF1	AFF3	AFF3
ACSL6	AFF3	AFF4	AFF4
ACTA2	AFF4	AKAP9	AKAP9
AF001548.5	AKAP9	AKT1	AKT1
AF001548.6	AKT1	ALDH2	AKT2
AFF1	AKT2	ALK	ALDH2
AFF3	ALDH2	AMER1	ALK
AFF4	ALK	APC	AMER1
AIP	AMER1	ARHGAP26	APC
AKAP9	APC	ARHGEF12	ARHGAP26
AKT1	ARHGAP26	ARID1A	ARHGEF12
AKT2	ARHGEF12	ARID1B	ARID1A
AKT3	ARID1A	ARID2	ARID1B
AL133247.2	ARID1B	ASXL1	ARID2
AL161450.1	ARID2	ATF1	ARNT
ALDH2	ARNT	ATIC	ASXL1
ALK	ASPSCR1	ATM	ATIC
AMER1	ASXL1	ATP1A1	ATM
ANKRD49	ATF1	ATP2B3	ATP1A1
AP000282.2	ATM	ATR	ATP2B3
AP000662.4	ATP1A1	ATRX	ATR
APC	ATP2B3	AXIN1	ATRX
AR	ATR	AXIN2	AXIN1
ARHGAP26	ATRX	BAP1	AXIN2
ARHGEF12	AXIN1	BCL10	BAP1
ARID1A	AXIN2	BCL11A	BCL10
ARID1B	BAP1	BCL11B	BCL11A
ARID2	BCL10	BCL3	BCL11B
ARID3A	BCL11A	BCL6	BCL2

ARID3B	BCL11B	BCL7A	BCL3
ARID4A	BCL2	BCL9	BCL6
ARID4B	BCL3	BCOR	BCL7A
ARID5A	BCL6	BCR	BCL9
ARID5B	BCL7A	BIRC3	BCOR
ARNT	BCL9	BLM	BCR
ASPSCR1	BCOR	BMPR1A	BIRC3
ASXL1	BCR	BRAF	BLM
ATF1	BIRC3	BRCA1	BMPR1A
ATIC	BLM	BRCA2	BRAF
ATM	BMPR1A	BRD4	BRCA1
ATR	BRAF	BRIP1	BRCA2
ATRX	BRCA1	BUB1B	BRD3
AURKA	BRCA2	C2orf44	BRD4
AVL9	BRD3	CACNA1D	BRIP1
AXIN2	BRD4	CALR	BTG1
BAP1	BRIP1	CAMTA1	BUB1B
BCL10	BUB1B	CANT1	C2orf44
BCL11A	C2orf44	CARD11	CACNA1D
BCL11B	CACNA1D	CARS	CALR
BCL2	CALR	CASC5	CAMTA1
BCL3	CAMTA1	CASP8	CANT1
BCL6	CANT1	CBL	CARD11
BCL7A	CARD11	CBLB	CARS
BCL9	CARS	CBLC	CASC5
BCOR	CASC5	CCDC6	CASP8
BCR	CASP8	CCNB1IP1	CBFA2T3
BIRC3	CBFA2T3	CD274	CBL
BIVM	CBFB	CD74	CBLB
BIVM-ERCC5	CBL	CD79A	CBLC
BLM	CBLB	CDC73	CCDC6
BMPR1A	CBLC	CDH1	CCNB1IP1
BRAF	CCDC6	CDH11	CCND1
BRCA1	CCNB1IP1	CDK12	CCND2
BRCA2	CCND2	CDK4	CCND3
BRD3	CCND3	CDK6	CCNE1
BRD4	CCNE1	CDKN1B	CD274
BRIP1	CD274	CDKN2A	CD74
BTG1	CD74	CDKN2C	CD79A
BUB1B	CD79A	CEP89	CD79B
C11orf65	CD79B	CHEK2	CDC73
C1orf147	CDC73	CHN1	CDH1
C4orf36	CDH1	CIC	CDH11
CANT1	CDH11	CLIP1	CDK12
CARD11	CDK12	CLP1	CDK4
CARS	CDK4	CLTC	CDK6
CARS-AS1	CDK6	CLTCL1	CDKN1B

CASC5	CDKN1B	CNOT3	CDKN2A
CBFA2T3	CDKN2A	CNTRL	CDKN2C
CBFB	CDKN2C	COL1A1	CDX2
CBL	CDX2	COL2A1	CEBPA
CBLB	CEBPA	CREB3L1	CEP89
CBLC	CEP89	CREB3L2	CHCHD7
CBR4	CHCHD7	CREBBP	CHEK2
CCDC140	CHEK2	CRTC1	CHIC2
CCDC6	CHIC2	CRTC3	CHN1
CCNB1IP1	CHN1	CSF3R	CIC
CCND1	CIC	CTNNB1	CLIP1
CCND2	CLIP1	CUX1	CLP1
CCND2-AS1	CLP1	CYLD	CLTCL1
CCND2-AS2	CLTC	DAXX	CNBP
CCND3	CLTCL1	DCTN1	CNOT3
CCNE1	CNOT3	DDB2	CNTRL
CD274	COL1A1	DDIT3	COL1A1
CD3EAP	COL2A1	DDX5	COL2A1
CD74	COX6C	DDX6	COX6C
CD79A	CREB1	DEK	CREB3L1
CD79B	CREB3L1	DICER1	CREB3L2
CDC42SE1	CREB3L2	DNM2	CREBBP
CDC73	CREBBP	DNMT3A	CRLF2
CDH1	CRLF2	EBF1	CRTC1
CDH11	CRTC1	ECT2L	CRTC3
CDH6	CRTC3	EGFR	CSF3R
CDK12	CSF3R	EIF3E	CTNNB1
CDK2AP2	CTNNB1	EIF4A2	CUX1
CDK4	CUX1	ELF4	CYLD
CDK6	CYLD	ELL	DAXX
CDK8	DAXX	ELN	DCTN1
CDKN1A	DCTN1	EML4	DDB2
CDKN1B	DDB2	EP300	DDIT3
CDKN2A	DDIT3	EPS15	DDX10
CDKN2B	DDX10	ERBB2	DDX5
CDKN2B-AS1	DDX5	ERBB3	DDX6
CDKN2C	DDX6	ERC1	DEK
CDKN2D	DEK	ERCC2	DICER1
CDX2	DICER1	ERCC3	DNM2
CDYL	DNM2	ERCC4	DNMT3A
CENPP	DNMT3A	ERCC5	EBF1
CEP128	EBF1	ERG	ECT2L
CERS2	ECT2L	ESR1	EGFR
CHCHD7	EGFR	ETNK1	EIF3E
CHD5	EIF3E	ETV1	EIF4A2
CHD6	EIF4A2	ETV4	ELF4
CHEK1	ELF4	ETV5	ELK4

CHEK2	ELK4	ETV6	ELL
CHIC2	ELL	EWSR1	ELN
CHN1	ELN	EXT1	EML4
CHRNA10	EML4	EZR	EP300
CHURC1-FNTB	EP300	FAM131B	EPS15
CIC	EPS15	FAM46C	ERBB2
CIITA	ERBB2	FANCA	ERBB3
CLP1	ERBB3	FANCD2	ERC1
CLTC	ERC1	FANCE	ERCC3
CLTCL1	ERCC3	FANCF	ERCC4
CMC4	ERCC4	FANCG	ERCC5
CNBP	ERCC5	FBXO11	ERG
CNTRL	ESR1	FBXW7	ESR1
COL1A1	ETV1	FCRL4	ETNK1
COX6C	ETV5	FGFR1	ETV1
CREB1	ETV6	FGFR2	ETV5
CREB3L1	EWSR1	FGFR3	ETV6
CREB3L2	EXT1	FGFR4	EWSR1
CREBBP	EXT2	FH	EXT1
CRKL	EZH2	FIP1L1	EXT2
CRLF2	EZR	FLCN	EZH2
CRTC1	FAM131B	FLI1	EZR
CRTC3	FAM46C	FLT3	FAM131B
CSF1R	FANCA	FLT4	FAM46C
CTB-4E7.1	FANCD2	FOXA1	FANCA
CTB-51J22.1	FANCE	FOXO1	FANCD2
CTB-58E17.3	FANCF	FOXO3	FANCE
CTB-58E17.9	FANCG	FOXO4	FANCF
CTC-215O4.4	FAS	FOXP1	FANCG
CTC-554D6.1	FBXO11	FUS	FAS
CTD-2135D7.2	FBXW7	GATA1	FBXO11
CTD-2196E14.3	FCRL4	GATA2	FBXW7
CTD-2196E14.5	FEV	GATA3	FCRL4
CTD-2207P18.1	FGFR1	GMPS	FGFR1
CTD-2267D19.2	FGFR2	GNA11	FGFR2
CTD-2301A4.5	FGFR3	GNAQ	FGFR3
CTD-2339L15.3	FGFR4	GNAS	FGFR4
CTD-2517M22.17	FH	GOLGA5	FH
CTD-2525I3.3	FHIT	GOPC	FHIT
CTD-2535L24.2	FLCN	GPC3	FIP1L1
CTD-2561B21.3	FLI1	GPHN	FLCN
CTD-2561B21.4	FLT3	GRIN2A	FLI1
CTD-3037G24.3	FLT4	H3F3B	FLT3
CTD-3065B20.3	FNBP1	HIP1	FLT4
CTD-3185P2.1	FOXA1	HIST1H3B	FNBP1
CTD-3214H19.4	FOXL2	HLA-A	FOXA1
CTNNB1	FOXO4	HLF	FOXL2

CYLD	FOXP1	HNF1A	FOXO1
CYP1B1	FUS	HNRNPA2B1	FOXO3
CYP1B1-AS1	GAS7	HOOK3	FOXO4
DAXX	GATA1	HOXA11	FOXP1
DCBLD1	GATA2	HOXA13	FUS
DDB2	GATA3	HSP90AA1	GAS7
DDIT3	GMPS	IDH1	GATA1
DDX10	GNA11	IDH2	GATA2
DDX5	GNAQ	IKBKB	GATA3
DDX6	GNAS	IL21R	GMPS
DDX60L	GOPC	IL6ST	GNA11
DECR1	GPC3	IL7R	GNAQ
DEK	GRIN2A	IRF4	GNAS
DGKG	H3F3A	ITK	GOPC
DHX8	H3F3B	JAK1	GPC3
DICER1	HERPUD1	JAK2	GRIN2A
DICER1-AS1	HEY1	JAK3	H3F3A
DNMT3A	HIP1	KAT6B	H3F3B
EBF1	HIST1H3B	KCNJ5	HERPUD1
EGFR	HLA-A	KDM5A	HEY1
EGFR-AS1	HNF1A	KDM5C	HIP1
EIF4A2	HNRNPA2B1	KDM6A	HIST1H3B
ELAC1	HOOK3	KDR	HLA-A
ELAC2	HOXA11	KIAA1598	HMGA2
ELF4	HOXA13	KIF5B	HNF1A
ELK4	HOXA9	KIT	HNRNPA2B1
ELL	HOXC13	KLK2	HOOK3
ELN	HOXD13	KMT2A	HOXA11
EML4	HRAS	KMT2C	HOXA13
EP300	HSP90AA1	KMT2D	HOXA9
EPCAM	HSP90AB1	KRAS	HOXD13
EPHA10	IDH1	KTN1	HRAS
EPHA3	IDH2	LASP1	HSP90AA1
EPHA5	IKBKB	LCK	HSP90AB1
EPHA6	IL2	LCP1	IDH1
EPHB6	IL21R	LHFP	IDH2
EPS15	IL6ST	LIFR	IKBKB
ERBB2	IL7R	LMNA	IKZF1
ERBB3	ITK	LPP	IL2
ERBB4	JAK1	LRIG3	IL6ST
ERC1	JAK2	LSM14A	IL7R
ERCC1	JAK3	MAF	ITK
ERCC2	JUN	MALAT1	JAK1
ERCC3	KAT6B	MALT1	JAK2
ERCC4	KCNJ5	MAML2	JAK3
ERCC5	KDM5A	MAP2K1	JAZF1
ERG	KDM5C	MAP2K2	JUN

ETV1	KDM6A	MAP2K4	KAT6B
ETV4	KDR	MAP3K13	KCNJ5
ETV5	KIAA1598	MDM2	KDM5A
ETV5-AS1	KIF5B	MDM4	KDM5C
ETV6	KIT	MDS2	KDM6A
EWSR1	KLF4	MECOM	KDR
EXT1	KLF6	MED12	KDSR
EXT2	KLK2	MEN1	KIAA1598
EZH2	KMT2A	MET	KIF5B
FAM46C	KMT2C	MITF	KIT
FAM71B	KMT2D	MKL1	KLF4
FANCA	KRAS	MLH1	KLF6
FANCC	KTN1	MLLT3	KLK2
FANCD2	LASP1	MLLT4	KMT2A
FANCD2OS	LCK	MN1	KMT2C
FANCE	LCP1	MPL	KMT2D
FANCG	LHFP	MSH2	KRAS
FAS	LIFR	MSH6	LASP1
FBXO11	LMNA	MSI2	LCK
FBXW7	LMO1	MSN	LCP1
FCGR2B	LMO2	MUC1	LHFP
FCGR2C	LPP	MUTYH	LIFR
FCGR3A	LRIG3	MYB	LMNA
FCRL4	LSM14A	MYC	LMO1
FEV	LYL1	MYCN	LMO2
FGF23	MAML2	MYD88	LRIG3
FGFR1	MAP2K1	MYH11	LSM14A
FGFR1OP	MAP2K2	MYH9	LYL1
FGFR2	MAP2K4	MYO5A	MAF
FGFR3	MAP3K13	MYOD1	MAFB
FGFR4	MAX	NAB2	MAML2
FH	MDM2	NACA	MAP2K1
FHIT	MDM4	NBN	MAP2K2
FHL1	MDS2	NCKIPSD	MAP2K4
FIP1L1	MECOM	NCOA1	MAP3K13
FKBP1B	MED12	NCOA2	MAX
FKBP9	MEN1	NCOR1	MDM2
FLCN	MET	NDRG1	MDM4
FLI1	MITF	NF1	MECOM
FLT1	MKL1	NF2	MED12
FLT3	MLH1	NFATC2	MEN1
FLT4	MLLT1	NIN	MET
FNBP1	MLLT3	NONO	MITF
FNTB	MLLT4	NOTCH1	MKL1
FOLR1	MN1	NOTCH2	MLH1
FOXO1	MNX1	NPM1	MLLT1
FOXO3	MPL	NRAS	MLLT10

FOXO4	MSH2	NRG1	MLLT3
FOXP1	MSH6	NSD1	MLLT4
FOXP1-AS1	MSN	NTRK1	MN1
FSTL3	MTCP1	NTRK3	MNX1
FUBP1	MUC1	NUMA1	MPL
FUS	MUTYH	NUP214	MSH2
GALNT3	MYB	NUP98	MSH6
GAS7	MYC	NUTM1	MSN
GATA1	MYCN	NUTM2A	MTCP1
GATA2	MYD88	NUTM2B	MUC1
GATA3	MYH11	OLIG2	MUTYH
GBGT1	MYH9	OMD	MYB
GGPS1	MYO5A	PALB2	MYC
GLMN	MYOD1	PAX3	MYCL
GMDS-AS1	NAB2	PAX5	MYCN
GMPS	NACA	PAX7	MYD88
GNA11	NBN	PAX8	MYH11
GNAQ	NCKIPSD	PBRM1	MYH9
GNAS	NCOA1	PCM1	MYO5A
GNAS-AS1	NCOA2	PCSK7	MYOD1
GNL3	NCOR1	PDCD1LG2	NAB2
GOLGA4	NDRG1	PDE4DIP	NACA
GOLGA5	NF1	PDGFRA	NBN
GOPC	NF2	PDGFRB	NCKIPSD
GP1BB	NFATC2	PER1	NCOA1
GPC3	NFE2L2	PHF6	NCOA2
GPHN	NFIB	PHOX2B	NCOR1
GSTM1	NFKB2	PICALM	NDRG1
GSTM2	NFKBIE	PIK3CA	NF1
GUCY1A2	NIN	PIK3R1	NF2
HDGF	NONO	PLCG1	NFATC2
HECW1	NOTCH1	PML	NFE2L2
HERPUD1	NOTCH2	PMS1	NFIB
HEY1	NPM1	PMS2	NFKB2
HIP1	NRAS	POLE	NFKBIE
HIST1H2BK	NRG1	POT1	NIN
HLF	NSD1	POU2AF1	NKX2-1
HMGA1	NT5C2	POU5F1	NONO
HMGA2	NTRK1	PPARG	NOTCH1
HMGN2P46	NTRK3	PPFIBP1	NOTCH2
HNF1A	NUMA1	PPP2R1A	NPM1
HNF1A-AS1	NUP214	PPP6C	NRAS
HNRNPA2B1	NUP98	PRDM1	NRG1
HOOK3	NUTM2A	PRDM16	NSD1
HOTAIR	NUTM2B	PRF1	NT5C2
HOTTIP	OLIG2	PRRX1	NTRK1
HOXA10-AS	OMD	PSIP1	NTRK3

HOXA11	P2RY8	PTCH1	NUMA1
HOXA13	PALB2	PTEN	NUP214
HOXA9	PAX3	PTPN11	NUP98
HOXC11	PAX5	PTPRB	NUTM1
HOXC13	PAX7	PTPRC	NUTM2A
HOXC13-AS	PAX8	PTPRK	OLIG2
HOXD10	PBRM1	PWWP2A	OMD
HOXD11	PCM1	RABEP1	P2RY8
HOXD13	PCSK7	RAD21	PALB2
HRAS	PDCD1LG2	RAD51B	PAX3
HSD17B3	PDE4DIP	RAF1	PAX5
HSD3B2	PDGFB	RALGDS	PAX7
HSP90AA1	PDGFRA	RANBP17	PAX8
HSP90AB1	PDGFRB	RANBP2	PBRM1
IDH1	PER1	RAP1GDS1	PBX1
IDH1-AS1	PHF6	RARA	PCM1
IDH2	PHOX2B	RB1	PCSK7
IGF1R	PICALM	RBM15	PDCD1LG2
IKBKE	PIK3CA	RECQL4	PDE4DIP
IKZF1	PIK3R1	REL	PDGFB
IL18BP	PIM1	RET	PDGFRA
IL2	PLAG1	RHOA	PDGFRB
IL21R	PLCG1	RHOH	PER1
IL21R-AS1	PML	RMI2	PHF6
IL6ST	PMS1	RNF213	PHOX2B
IL7R	PMS2	RNF43	PICALM
INSRR	POLE	ROS1	PIK3CA
IRF4	POT1	RPL10	PIK3R1
ITK	POU2AF1	RPL22	PIM1
JAK1	POU5F1	RPN1	PLAG1
JAK2	PPARG	RSPO2	PLCG1
JAK3	PPFIBP1	RUNDC2A	PML
JAZF1	PPP2R1A	RUNX1	PMS1
JAZF1-AS1	PRCC	RUNX1T1	PMS2
JUP	PRDM1	SBDS	POLE
KAT6A	PRDM16	SDHAF2	POT1
KAT6B	PRF1	SDHC	POU2AF1
KCNK12	PRKAR1A	SETBP1	POU5F1
KDM5A	PSIP1	SETD2	PPARG
KDM5C	PTCH1	SF3B1	PPFIBP1
KDM6A	PTEN	SFPQ	PPP2R1A
KDR	PTPN11	SH2B3	PPP6C
KDSR	PTPRB	SH3GL1	PRCC
KEAP1	PTPRC	SLC34A2	PRDM1
KIAA1549	PTPRK	SLC45A3	PRDM16
KIF1B	PWWP2A	SMAD4	PRF1
KIT	RAC1	SMARCA4	PRKAR1A

KL	RAD21	SMARCB1	PSIP1
KLF6	RAD51B	SMARCD1	PTCH1
KLK2	RAF1	SMO	PTEN
KMT2A	RALGDS	SND1	PTPN11
KMT2C	RANBP17	SPECC1	PTPRB
KMT2D	RANBP2	SPEN	PTPRC
KRAS	RAP1GDS1	SPOP	PTPRK
KRT17	RARA	SRGAP3	PWWP2A
KRT18P62	RB1	SS18	RAC1
KTN1	RECQL4	SS18L1	RAD21
KTN1-AS1	REL	STAG2	RAD51B
LASP1	RET	STAT3	RAF1
LCK	RHOA	STAT5B	RALGDS
LCP1	RHOH	STAT6	RANBP17
LHFP	RNF213	STIL	RANBP2
LIFR	RNF43	STK11	RAP1GDS1
LIFR-AS1	ROS1	SUFU	RARA
LINC00094	RPL10	SUZ12	RB1
LMO1	RPL22	SYK	RECQL4
LMO2	RPL5	TBL1XR1	REL
LPP	RPN1	TBX3	RET
LPP-AS1	RSPO2	TCF12	RHOA
LPP-AS2	RSPO3	TCF3	RHOH
LRP5	RUNDC2A	TCF7L2	RMI2
LRRFIP2	RUNX1	TCL1A	RNF213
LSM3	RUNX1T1	TERT	RNF43
LTBP2	SBDS	TET1	ROS1
LTBP3	SDC4	TET2	RPL10
LYL1	SDHAF2	TFE3	RPL22
MAD2L1BP	SDHB	TFEB	RPL5
MAF	SDHC	TFPT	RPN1
MALT1	SDHD	TFRC	RSPO2
MAML2	SEPT6	THRAP3	RSPO3
MAP2K1	SEPT9	TNFAIP3	RUNDC2A
MAP2K2	SET	TOP1	RUNX1
MAP2K4	SETBP1	TP53	RUNX1T1
MAP3K1	SETD2	TPM3	SBDS
MAP3K8	SF3B1	TPR	SDC4
MAP3L1	SFPQ	TRAF7	SDHAF2
MARCH9	SH2B3	TRIM24	SDHB
MAX	SH3GL1	TRIM27	SDHC
MC1R	SLC34A2	TRIM33	SDHD
MCL1	SLC45A3	TRIP11	SEPT6
MDM2	SMAD4	TRRAP	SEPT9
MDM4	SMARCA4	TSC1	SET
MDS2	SMARCB1	TSC2	SETBP1
MECOM	SMARCD1	TSHR	SETD2

MED12	SMO	TTL	SF3B1
MED7	SND1	UBR5	SFPQ
MEN1	SOCS1	VHL	SH2B3
MET	SOX2	VTI1A	SH3GL1
METTL21A	SPECC1	WAS	SLC34A2
MFSD11	SPEN	WHSC1	SLC45A3
MFSD2B	SPOP	WHSC1L1	SMAD4
MFSD4	SRGAP3	WIF1	SMARCA4
MITF	SRSF2	WRN	SMARCB1
MKL1	SRSF3	WT1	SMARCD1
MLF1	SS18	WWTR1	SMO
MLH1	SSX1	XPC	SND1
MLLT1	STAG2	XPO1	SOCS1
MLLT10	STAT3	YWHAE	SOX2
MLLT11	STAT5B	ZCCHC8	SPEN
MLLT3	STAT6	ZNF198	SPOP
MLLT4	STIL	ZNF278	SRGAP3
MLLT6	STK11	ZNF331	SRSF2
MN1	STRN	ZNF384	SRSF3
MNX1	SUFU	ZNF521	SS18
MNX1-AS2	SUZ12	ZRSR2	SSX1
MPL	SYK		STAG2
MPRIP	TAL1		STAT3
MRE11A	TBL1XR1		STAT5B
MSH2	TBX3		STAT6
MSH6	TCEA1		STIL
MSI2	TCF12		STK11
MSN	TCF3		STRN
MTCP1	TCF7L2		SUFU
MTOR	TCL1A		SUZ12
MTOR-AS1	TERT		SYK
MTUS2	TET1		TAL1
MTUS2-AS1	TET2		TBL1XR1
MUC1	TFE3		TBX3
MUTYH	TFEB		TCEA1
MYB	TFPT		TCF12
MYB-AS1	TFRC		TCF3
MYC	THRAP3		TCF7L2
MYCL	TLX1		TCL1A
MYCN	TNFAIP3		TCL6
MYCNOS	TNFRSF17		TERT
MYD88	TOP1		TET2
MYH11	TP53		TFE3
MYH9	TPM3		TFEB
MYOC	TPM4		TFPT
NACA	TPR		TFRC
NBN	TRAF7		THRAP3

NCKIPSD
NCOA1
NCOA2
NCOA4
NDE1
NDRG1
NDUFA3
NF1
NF2
NFE2L2
NFIB
NFKB2
NIN
NKX2-1
NKX2-1-AS1
NONO
NOTCH1
NOTCH2
NOTCH3
NOTCH4
NPM1
NR4A3
NRAS
NSD1
NTRK1
NTRK2
NTRK3
NTRK3-AS1
NUMA1
NUP214
NUP88
NUP98
NUTM1
NUTM2A
NUTM2A-AS1
NUTM2B
OMD
OPTN
OSMR
P2RY8
PAFAH1B2
PAK6
PAK7
PALB2
PALLD
PATZ1
PAX2

TRIM24
TRIM27
TRIM33
TRIP11
TRRAP
TSC1
TSC2
TSHR
TTL
U2AF1
UBR5
USP6
VHL
VTI1A
WAS
WHSC1
WHSC1L1
WIF1
WRN
WT1
WWTR1
XPA
XPC
XPO1
ZCCHC8
ZNF198
ZNF278
ZNF384
ZNF521
ZRSR2

TMPRSS2
TNFAIP3
TNFRSF17
TOP1
TP53
TPM3
TPR
TRAF7
TRIM24
TRIM27
TRIM33
TRIP11
TRRAP
TSC1
TSC2
TSHR
U2AF1
UBR5
USP6
VHL
VTI1A
WAS
WHSC1
WHSC1L1
WIF1
WRN
WT1
WWTR1
XPA
XPC
XPO1
YWHAE
ZBTB16
ZCCHC8
ZNF198
ZNF278
ZNF331
ZNF384
ZNF521
ZRSR2

PAX3
PAX5
PAX6
PAX7
PAX8
PAX8-AS1
PBRM1
PBX1
PCM1
PCSK7
PDCD1LG2
PDE4DIP
PDGFB
PDGFRA
PDGFRB
PDPK1
PER1
PGAP3
PHF6
PHOX2B
PICALM
PIK3CA
PIK3R1
PIM1
PLAG1
PLK1
PML
PMS1
PMS2
POU2AF1
POU5F1
PPARG
PPP2R1A
PRCC
PRDM1
PRDM16
PRF1
PRG4
PRKAR1A
PRKDC
PRRX1
PSIP1
PTCH1
PTEN
PTK2
PTK2B
PTPN11

PTPRD
PTRH2
RABEP1
RAD51B
RAF1
RALGDS
RANBP17
RAP1GDS1
RARA
RB1
RBM15
RBM34
RCN1
RECQL4
REL
RET
RHOH
RICTOR
RMDN2
RMI2
RNASEL
RNF170
RNF207
ROS1
RP11-1070N10.6
RP11-114N19.3
RP11-1167A19.2
RP11-119F19.2
RP11-120M18.2
RP11-126O1.4
RP11-126O1.5
RP11-131L23.1
RP11-133K1.2
RP11-143H17.1
RP11-145E5.5
RP11-148G20.1
RP11-152K4.2
RP11-162P23.2
RP11-164H13.1
RP11-166B2.1
RP11-166D19.1
RP11-169K16.9
RP11-191G24.1
RP11-201K10.3
RP11-20I23.6
RP11-20I23.8
RP11-211G3.3

RP11-227F19.1	
RP11-23F23.2	
RP11-240L7.4	
RP11-242D8.1	
RP11-25K21.1	
RP11-25K21.6	
RP11-285E9.6	
RP11-286H14.8	
RP11-286N22.8	
RP11-286O18.1	
RP11-297B17.3	
RP11-298J23.5	
RP11-318A15.7	
RP11-31L23.3	
RP11-323J4.1	
RP11-327F22.1	
RP11-327F22.4	
RP11-350N15.4	
RP11-354M1.2	
RP11-355B11.2	
RP11-356I2.4	
RP11-359B12.2	
RP11-363L24.3	
RP11-366L20.2	
RP11-370K11.1	
RP11-374P20.4	
RP1-137K2.2	
RP11-380O24.1	
RP11-383G6.3	
RP11-387D10.2	
RP11-392O1.4	
RP11-39K24.2	
RP11-420A6.2	
RP11-434D12.1	
RP11-435O5.5	
RP11-45M22.4	
RP11-461L13.2	
RP11-475N22.4	
RP11-513D5.2	
RP11-513D5.5	
RP11-517A5.7	
RP11-517H2.6	
RP11-517O13.3	
RP11-519G16.3	
RP11-530I17.1	
RP11-544A12.4	
RP11-544A12.8	

RP11-573D15.9
RP11-57H14.2
RP11-598P20.5
RP11-599B13.6
RP11-603J24.9
RP11-619L19.1
RP11-635L1.3
RP11-64D24.2
RP11-654A16.1
RP11-654A16.3
RP11-6B6.3
RP1-170O19.20
RP11-729L2.2
RP11-72M17.1
RP11-744N12.3
RP11-770J1.3
RP11-77G23.2
RP11-794P6.2
RP11-807H22.7
RP11-830F9.5
RP11-830F9.6
RP11-840I19.3
RP11-848P1.5
RP11-849H4.4
RP11-876N24.2
RP11-896J10.3
RP1-18D14.7
RP11-936I5.1
RP1-1J6.2
RP1-309F20.3
RP1-85F18.5
RP1-85F18.6
RP3-395M20.8
RP3-400N23.6
RP3-416J7.4
RP4-761J14.8
RP4-791M13.4
RP4-791M13.5
RP5-951N9.1
RP5-951N9.2
RPL21P122
RPL22
RPL6
RPN1
RPSAP52
RPTOR
RRM1

RUNX1
RUNX1T1
SARDH
SBDS
SDCCAG8
SDHAF2
SDHB
SDHC
SDHD
SENCR
SEPT5
SEPT6
SEPT9
SET
SETD2
SETD5-AS1
SETDB1
SF3B1
SF3B2
SFPQ
SH2D2A
SH3GL1
SLC22A23
SLC30A4
SLC45A3
SMAD2
SMAD3
SMAD4
SMARCA4
SMARCB1
SMIM4
SMO
SNAPC3
SNAPC5
SNX29
SOCS1
SOX2-OT
SPECC1
SPEN
SRC
SRD5A2
SRGAP3
SRGAP3-AS1
SRSF3
SS18
SS18L1
SSX1

SSX2
SSX4
STAMBPL1
STAT3
STIL
STK11
STX11
STXBP2
SUFU
SUZ12
SYK
TAF15
TAL1
TAX1BP1
TCEA1
TCEA1P2
TCF12
TCF19
TCF3
TCF4
TCF7L2
TCL1A
TCL6
TERT
TET1
TET2
TFE3
TFEB
TFG
TFPT
TFRC
TGFBR2
THRAP3
TLX1
TLX3
TMEM127
TMPRSS2
TNFAIP3
TNFRSF14
TNFRSF17
TOE1
TOP1
TOP2A
TP53
TPM3
TPM4
TPR

TRIM24
TRIM27
TRIM33
TRIP11
TSC1
TSC2
TSHR
TSPAN31
TTL
TYK2
TYW1
U2AF1
ULK4
UNC13D
USP6
UTY
VHL
VPS13B
VTI1A
WAS
WDR36
WHSC1
WHSC1L1
WIF1
WRAP53
WRN
WT1
XPA
XPC
XPO1
YPEL4
YWHAE
ZBTB16
ZMYM2
ZNF232
ZNF276
ZNF280D
ZNF331
ZNF384
ZNF521
ZNF668
ZRSR2

Table S2A. The performances of the four algorithms in ten experiments, checked agianst the reported mutations in TCGA EC paper; GATKcan used the cutoffs trained by ten randomly sampled EC tumors in each repeat.

Repeat		1	2	3	4	5	6	7	8	9	10	**Mean**	**SE**
No. of called variants		62,025	61,938	60,967	62,117	60,760	61,263	61,442	61,310	61,948	61,302	61,507.2	473.2
No. of somatic variants		2,067	2,128	2,150	2,110	2,038	2,125	2,069	2,083	2,122	2,124	2,101.6	35.3
GATK	TPR	88.2%	88.3%	89.6%	88.2%	87.2%	88.0%	88.0%	88.0%	88.2%	88.0%	88.2%	0.6%
	cFPR	65.1%	65.1%	65.4%	65.2%	65.2%	65.2%	64.9%	65.2%	65.1%	65.1%	65.2%	0.1%
	precision	13.0%	13.0%	12.5%	12.9%	11.7%	12.5%	12.5%	12.6%	13.1%	12.6%	12.6%	0.4%
	MCC	14.7%	14.8%	15.1%	14.6%	13.4%	14.3%	14.4%	14.4%	14.7%	14.4%	14.5%	0.4%
	TPR†	98.7%	98.8%	98.8%	98.8%	98.8%	98.8%	98.7%	98.7%	98.8%	98.8%	98.8%	0.0%
	cFPR†	81.5%	81.1%	81.0%	80.5%	80.6%	80.4%	80.9%	81.0%	81.1%	81.4%	81.0%	0.4%
	precision†	54.3%	54.9%	54.9%	54.8%	54.1%	54.8%	54.5%	54.6%	55.1%	54.6%	54.7%	0.3%
	MCC†	28.7%	29.3%	29.5%	29.9%	29.7%	29.9%	29.3%	29.3%	29.5%	29.0%	29.4%	0.4%
GATKcan	TPR	95.3%	95.2%	98.7%	94.9%	97.0%	98.3%	94.7%	98.5%	93.5%	94.7%	96.1%	1.9%
	cFPR	11.5%	11.4%	14.2%	10.8%	13.4%	14.2%	10.8%	14.0%	10.7%	10.8%	12.2%	1.6%
	precision	47.9%	48.1%	42.0%	49.0%	41.9%	42.4%	48.1%	42.9%	49.3%	48.5%	46.0%	3.2%
	MCC	63.0%	63.1%	59.4%	63.8%	58.9%	59.6%	63.1%	60.0%	63.4%	63.4%	61.8%	2.0%
	TPR†	98.1%	98.4%	99.4%	98.0%	98.9%	99.3%	98.0%	99.4%	98.0%	98.0%	98.6%	0.6%
	cFPR†	5.6%	5.4%	6.4%	5.7%	5.4%	6.3%	5.3%	5.9%	5.9%	5.5%	5.7%	0.4%
	precision†	94.5%	94.7%	93.9%	94.5%	94.6%	94.0%	94.8%	94.3%	94.4%	94.6%	94.4%	0.3%
	MCC†	92.6%	93.0%	93.2%	92.4%	93.5%	93.2%	92.7%	93.6%	92.2%	92.5%	92.9%	0.5%
VarScan2	TPR†	99.6%	99.6%	99.6%	99.6%	99.6%	99.6%	99.6%	99.6%	99.6%	99.6%	99.6%	0.0%
	cFPR†	60.9%	60.3%	60.4%	60.1%	59.6%	60.6%	60.0%	60.1%	62.1%	60.7%	60.5%	0.7%
	precision†	61.6%	62.3%	62.2%	62.1%	61.6%	61.9%	62.0%	62.1%	61.8%	62.0%	62.0%	0.2%
	MCC†	48.4%	49.1%	49.1%	49.1%	49.2%	48.8%	49.2%	49.1%	47.8%	48.7%	48.9%	0.5%
MuTect	TPR†	94.4%	94.4%	94.2%	94.2%	94.2%	94.3%	94.2%	94.3%	94.3%	94.4%	94.3%	0.1%
	cFPR†	38.1%	37.9%	37.7%	37.6%	36.8%	37.9%	38.0%	38.1%	39.0%	38.2%	37.9%	0.5%
	precision†	70.8%	71.3%	71.3%	71.2%	71.1%	71.1%	70.9%	71.0%	70.9%	71.1%	71.1%	0.2%
	MCC†	59.4%	59.7%	59.6%	59.6%	60.1%	59.5%	59.3%	59.4%	58.7%	59.4%	59.5%	0.4%

†adjusted TPR, cFPR, MCC and precision.

Table S2B. The six thresholds of GATKcan trained by randomly sampled 44 TCGA EC tumors and performances of GATKcan in the ten training experiments.

Repeat		1	2	3	4	5	6	7	8	9	10
α		0.5	0.5	0.5	0.3	0.3	0.4	0.4	0.3	0.5	0.5
dNM		991.1	988.1	990.5	985.6	984.3	981.5	989.3	982.3	987.9	989.1
FS		46.1	43.6	45.8	49.3	51.8	50.9	45.7	55.2	47.0	45.6
MQ		50	49.5	39.2	48.2	48	50	39.9	50	49.9	39.9
MQRankSum		-7.9	-7.9	-10.1	-10.1	-10.4	-9.6	-9.0	-8.4	-10.2	-9.6
QD		0.1	0.1	0.1	0.1	0.1	0.1	0.1	0.2	0.1	0.0
Mann-Whitney test (P value)		0.0100	0.0100	0.0100	0.0100	0.0090	0.0100	0.0820	0.0100	0.0100	0.0100
Training	TPR	98.58	98.10	99.29	98.59	98.88	98.66	99.30	99.60	98.22	99.17
	cFPR	10.68	10.65	14.32	12.24	11.58	10.76	13.99	11.91	10.54	14.29

Table S2C. The four thresholds of GATKcan trained by ~18% of 539 reported indels and 112 non-indels (from 241 TCGA EC tumors), and performances of GATKcan in the ten training experiments.

Repeat		1	2	3	4	5	6	7	8	9	10
α		0.5	0.5	0.3	0.5	0.5	0.6	0.3	0.3	0.5	0.5
dNM		753.4	405.0	581.6	395.2	658.8	464.0	397.6	619.2	566.9	633.6
FS		86.3	133.3	91.6	136.6	102.7	121.0	95.1	99.1	135.7	139.9
QD		0.28	0.43	0.28	0.76	0.4	0.18	0.23	0.41	0.49	0.81
Mann-Whitney test (P value)		0.0550	0.0900	0.0550	0.0640	0.0134	0.0370	0.0271	0.0460	0.1000	0.0033
Training	TPR	100.00	100.00	100.00	100.00	100.00	100.00	100.00	100.00	100.00	100.00
	cFPR	0.00	0.00	0.00	0.00	0.00	0.00	0.00	0.00	0.00	0.00

Table S2D. The performances of the four algorithms in ten experiments, checked agianst the reported mutations in TCGA EC paper; GATKcan used the cutoffs trained by 44 randomly sampled EC tumors in each repeat.

Repeat		1	2	3	4	5	6	7	8	9	10	**Mean**	**SE**
No. of called variants		52,129	52,672	51,805	50,206	53,059	52,100	52,265	52,713	52,972	52,984	52,290.5	850.1
No. of somatic variants		1,337	1,825	1,933	1,581	1,893	1,795	1,904	1,364	1,861	1,915	1,740.8	229.0
GATK	TPR	86.7%	87.3%	90.3%	86.6%	88.4%	87.8%	89.4%	87.2%	88.0%	89.7%	88.1%	1.3%
	cFPR	64.8%	65.3%	65.2%	64.3%	65.3%	64.8%	65.5%	64.7%	65.1%	65.4%	65.0%	0.4%
	precision	12.2%	12.7%	13.0%	10.4%	13.5%	12.4%	12.9%	12.6%	12.9%	13.6%	12.6%	0.9%
	MCC	13.6%	14.0%	15.9%	12.7%	15.1%	14.3%	15.2%	14.1%	14.6%	15.8%	14.5%	1.0%
	TPR†	98.3%	99.0%	99.0%	98.8%	98.9%	99.1%	99.0%	98.3%	99.2%	98.8%	98.8%	0.3%
	cFPR†	75.5%	81.8%	81.7%	79.6%	80.1%	80.9%	81.9%	77.0%	80.9%	82.9%	80.2%	2.3%
	precision†	54.2%	54.9%	55.9%	54.2%	56.3%	54.6%	54.9%	54.5%	55.2%	55.3%	55.0%	0.7%
	MCC†	33.1%	29.3%	29.6%	30.7%	30.8%	30.2%	29.1%	32.1%	30.7%	27.7%	30.3%	1.5%
GATKcan	TPR	94.1%	95.1%	99.1%	95.3%	96.3%	94.2%	98.6%	94.2%	94.7%	99.1%	96.1%	2.1%
	cFPR	10.9%	11.8%	14.8%	11.7%	12.0%	10.9%	14.3%	10.7%	11.2%	14.7%	12.3%	1.6%
	precision	47.2%	46.7%	41.9%	41.2%	48.2%	47.5%	42.8%	48.5%	48.2%	43.6%	45.6%	2.9%
	MCC	62.2%	62.0%	59.4%	58.3%	63.4%	62.4%	59.9%	63.1%	63.0%	60.6%	61.4%	1.8%
	TPR†	97.0%	98.7%	99.5%	98.8%	98.4%	98.1%	99.6%	96.8%	98.3%	99.5%	98.5%	1.0%
	cFPR†	4.4%	6.0%	7.2%	6.1%	6.3%	5.8%	6.9%	4.7%	6.4%	6.9%	6.1%	0.9%
	precision†	95.2%	94.3%	93.5%	94.0%	94.3%	94.3%	93.6%	95.1%	93.9%	93.7%	94.2%	0.6%
	MCC†	92.5%	92.8%	92.6%	92.8%	92.4%	92.3%	92.9%	92.1%	92.0%	92.9%	92.5%	0.3%
VarScan2	TPR†	99.5%	99.7%	99.6%	99.5%	99.6%	99.7%	99.6%	99.5%	99.7%	99.6%	99.6%	0.1%
	cFPR†	50.8%	62.2%	60.9%	57.2%	63.8%	61.3%	62.4%	52.7%	61.7%	63.0%	59.6%	4.5%
	precision†	64.0%	61.7%	63.1%	62.4%	62.0%	61.5%	61.7%	63.9%	61.9%	62.1%	62.4%	0.9%
	MCC†	55.5%	47.7%	49.0%	50.9%	46.6%	48.3%	47.4%	54.3%	48.2%	47.3%	49.5%	3.1%
MuTect	TPR†	92.6%	94.6%	94.1%	95.3%	93.9%	94.4%	94.7%	92.3%	94.7%	94.2%	94.1%	0.9%
	cFPR†	26.5%	39.6%	39.5%	34.1%	40.5%	38.9%	39.9%	28.6%	39.0%	41.1%	36.8%	5.2%
	precision†	76.0%	70.6%	71.4%	72.7%	70.8%	70.4%	70.5%	75.2%	70.9%	70.4%	71.9%	2.1%
	MCC†	66.9%	58.7%	58.3%	63.8%	57.1%	58.8%	58.4%	64.8%	59.3%	56.9%	60.3%	3.5%

[†] adjusted TPR, cFPR, MCC and precision.

Table S3. The performances of GATKcan in ten repeats, checked agianst the reported mutations in TCGA BC paper. In each repeat, only the set of trained cutoffs were different; no indels was in the called variants of BC .

The kth set of Cutoffs		1	2	3	4	5	6	7	8	9	10	**Mean**	**SE**
GATKcan (trained by 10 EC tumors)	TPR	70.6%	70.6%	70.9%	70.6%	70.7%	70.8%	70.6%	70.8%	70.0%	70.6%	**70.6%**	**0.2%**
	cFPR	3.2%	3.0%	3.4%	3.1%	3.5%	3.4%	3.2%	3.4%	3.1%	3.2%	**3.3%**	**0.2%**
	precision	44.9%	46.4%	43.2%	45.2%	42.6%	43.0%	44.3%	43.5%	45.0%	44.6%	**44.3%**	**1.2%**
	MCC	54.4%	55.3%	53.3%	54.5%	52.8%	53.2%	54.0%	53.5%	54.2%	54.1%	**53.9%**	**0.7%**
	TPR†	99.3%	99.3%	100.0%	99.3%	99.3%	100.0%	99.3%	100.0%	99.3%	99.3%	**99.5%**	**0.3%**
	cFPR†	2.3%	2.3%	5.1%	2.9%	3.9%	3.5%	2.3%	3.2%	3.9%	2.3%	**3.2%**	**0.9%**
	precision†	95.4%	95.4%	90.2%	94.2%	92.4%	93.0%	95.4%	93.6%	92.4%	95.4%	**93.7%**	**1.8%**
	MCC†	96.1%	96.1%	92.5%	95.1%	93.8%	94.7%	96.1%	95.2%	93.8%	96.1%	**95.0%**	**1.3%**
GATKcan (trained by 44 EC tumors)	TPR	70.6%	70.6%	70.9%	70.6%	70.6%	70.6%	70.9%	70.6%	70.6%	70.9%	**70.7%**	**0.1%**
	cFPR	3.2%	3.2%	3.8%	3.3%	3.4%	3.2%	3.4%	3.2%	3.2%	3.8%	**3.4%**	**0.2%**
	precision	44.6%	44.4%	40.5%	43.8%	43.4%	44.5%	43.2%	44.5%	44.4%	40.6%	**43.4%**	**1.6%**
	MCC	54.1%	54.0%	51.4%	53.6%	53.4%	54.1%	53.3%	54.1%	54.0%	51.5%	**53.4%**	**1.0%**
	TPR†	99.3%	99.3%	100.0%	99.3%	99.3%	99.3%	100.0%	99.3%	99.3%	100.0%	**99.5%**	**0.3%**
	cFPR†	4.5%	4.5%	5.1%	4.5%	4.5%	4.5%	5.1%	4.5%	4.5%	5.1%	**4.7%**	**0.3%**
	precision†	91.3%	91.3%	90.2%	91.3%	91.3%	91.3%	90.2%	91.3%	91.3%	90.2%	**91.0%**	**0.5%**
	MCC†	92.8%	92.8%	92.5%	92.8%	92.8%	92.8%	92.5%	92.8%	92.8%	92.5%	**92.7%**	**0.1%**

[†]adjusted TPR, cFPR, MCC and precision.

Table S4. The performances of GATKcan in tenrepeats, checked agianst the reported mutations in TCGA OC paper. In each repeat, only the trained cutoffs were different.

The kth set of Cutoffs		1	2	3	4	5	6	7	8	9	10	**Mean**	**SE**
GATKcan (trained by 10 EC tumors and ~9% indels)	TPR	90.0%	87.8%	87.8%	90.0%	90.0%	87.8%	90.0%	87.8%	89.8%	90.0%	**89.1%**	**1.1%**
	cFPR	4.7%	5.0%	5.0%	4.8%	5.5%	5.2%	4.6%	5.1%	4.8%	4.9%	**5.0%**	**0.3%**
	precision	31.7%	30.1%	30.2%	31.2%	28.6%	29.3%	32.1%	29.6%	31.5%	31.0%	**30.5%**	**1.1%**
	MCC	51.7%	49.7%	49.8%	51.3%	48.9%	48.9%	52.1%	49.2%	51.5%	51.1%	**50.4%**	**1.2%**
	TPR†	98.2%	98.2%	98.2%	98.2%	98.2%	98.2%	98.2%	98.2%	98.2%	98.2%	**98.2%**	**0.0%**
	cFPR†	3.3%	3.3%	4.9%	3.3%	6.6%	4.9%	3.3%	4.9%	3.3%	3.3%	**4.1%**	**1.2%**
	precision†	93.2%	93.2%	90.2%	93.2%	87.3%	90.2%	93.2%	90.2%	93.2%	93.2%	**91.7%**	**2.1%**
	MCC†	93.6%	93.6%	91.3%	93.6%	89.0%	91.3%	93.6%	91.3%	93.6%	93.6%	**92.5%**	**1.6%**
GATKcan (trained by 44 EC tumors and ~18% indels)	TPR	90.0%	90.0%	90.0%	90.0%	90.0%	90.0%	87.8%	90.0%	90.0%	90.0%	**89.8%**	**0.7%**
	cFPR	4.6%	5.2%	5.1%	5.3%	4.9%	5.2%	5.5%	4.8%	4.9%	5.6%	**5.1%**	**0.3%**
	precision	32.5%	29.6%	30.0%	29.4%	31.0%	29.7%	28.0%	31.5%	31.0%	28.2%	**30.1%**	**1.4%**
	MCC	52.5%	49.9%	50.2%	49.6%	51.2%	49.9%	47.7%	51.6%	51.1%	48.5%	**50.2%**	**1.4%**
	TPR†	98.2%	98.2%	98.2%	98.2%	98.2%	98.2%	98.2%	98.2%	98.2%	98.2%	**98.2%**	**0.0%**
	cFPR†	3.3%	3.3%	4.9%	4.1%	4.1%	3.3%	4.9%	3.3%	3.3%	4.9%	**3.9%**	**0.7%**
	precision†	93.2%	93.2%	90.2%	91.7%	91.7%	93.2%	90.2%	93.2%	93.2%	90.2%	**92.0%**	**1.4%**
	MCC†	93.6%	93.6%	91.3%	92.5%	92.5%	93.6%	91.3%	93.6%	93.6%	91.3%	**92.7%**	**1.1%**

†adjusted TPR, cFPR, MCC and precision.

Table S5. The performances of GATKcan in ten repeats, checked agianst the reported mutations in TCGA cutaneous melanoma paper. In each repeat, only the set of trained cutoffs were different; no indels was in the called variants of Melanoma.

The kth set of Cutoffs		1	2	3	4	5	6	7	8	9	10	**Mean**	**SE**
GATKcan (trained by 10 EC tumors)	TPR	98.7%	97.9%	99.1%	98.6%	98.9%	99.1%	98.6%	99.1%	98.2%	98.6%	**98.7%**	**0.4%**
	cFPR	4.6%	3.9%	4.1%	4.6%	4.7%	4.1%	4.6%	4.0%	4.7%	4.6%	**4.4%**	**0.3%**
	precision	85.3%	87.2%	86.8%	85.2%	85.1%	86.8%	85.3%	87.0%	85.2%	85.3%	**85.9%**	**0.9%**
	MCC	89.4%	90.2%	90.7%	89.2%	89.4%	90.7%	89.3%	90.8%	89.0%	89.3%	**89.8%**	**0.7%**
	TPR†	99.0%	99.1%	99.6%	98.9%	99.2%	99.6%	99.0%	99.4%	98.9%	99.0%	**99.2%**	**0.3%**
	cFPR†	8.4%	8.4%	10.3%	7.7%	9.3%	10.3%	7.7%	10.3%	7.7%	7.7%	**8.8%**	**1.2%**
	precision†	97.4%	97.4%	96.8%	97.6%	97.1%	96.8%	97.6%	96.8%	97.6%	97.6%	**97.3%**	**0.4%**
	MCC†	92.4%	92.5%	92.2%	92.6%	92.1%	92.4%	92.7%	91.9%	92.6%	92.7%	**92.4%**	**0.3%**
GATKcan (trained by 44 EC tumors)	TPR	98.6%	98.7%	99.3%	98.9%	98.9%	98.6%	99.1%	98.6%	98.6%	99.3%	**98.9%**	**0.3%**
	cFPR	4.7%	4.7%	4.8%	4.7%	4.7%	4.7%	4.1%	4.7%	4.7%	4.8%	**4.7%**	**0.2%**
	precision	85.2%	85.2%	84.9%	85.1%	85.1%	85.2%	86.9%	85.2%	85.2%	84.9%	**85.3%**	**0.6%**
	MCC	89.3%	89.3%	89.5%	89.3%	89.3%	89.2%	90.8%	89.2%	89.3%	89.5%	**89.5%**	**0.5%**
	TPR†	99.0%	99.1%	99.6%	99.2%	99.2%	99.0%	99.6%	99.0%	99.0%	99.6%	**99.2%**	**0.3%**
	cFPR†	7.7%	8.2%	10.3%	8.6%	8.6%	7.7%	10.3%	7.9%	7.7%	10.3%	**8.7%**	**1.1%**
	precision†	97.6%	97.5%	96.8%	97.3%	97.3%	97.6%	96.8%	97.5%	97.6%	96.8%	**97.3%**	**0.3%**
	MCC†	92.7%	92.7%	92.4%	92.5%	92.5%	92.7%	92.2%	92.5%	92.7%	92.2%	**92.5%**	**0.2%**

†adjusted TPR, cFPR, MCC and precision.

Table S6. False positive rate (in Mb^{-1}) of GATK and GATKcan in detecting mutations of randomly sampled 10% reference sites of cancer genes in four cancers; GATKacn used the first set of trained cutoffs by ten EC tumors.

Cancer	No. of samples	Reference sites (Mb)	FPR (Mb^{-1})	
			GATK	GATKcan
EC	241	60.4	313	189
BC	503	84.9	184	94
OC	215	29.7	126	85
Melanoma	342	46.9	101	64

Note1

The settings of the parameters in PSO

The upper/lower bound and step size for each statistic

Statistics	Exome-seq		Step size
	Lower bound	Upper bound	
dNB	0	1000	1
FS	0	60	1
MQ	30	50	1
MQRankSum	-12.5	-2.5	0.1
QD	0	2	0.1
p-value of Mann-Whitney test	10^{-1}	10^{-6}	10^{-1}

The number of seed points = 4100

The number of generations = 500

Fitness function = $\alpha * (1 - FPR) + (1 - \alpha) * (TPR)$

The collected reported mutations in 19 TCGA cancer types (by Jan., 2017), where the cancer types are tabulated as follows.

TCGA project	Cancer type
ACC	Adrenocortical carcinoma
BLCA	Bladder Urothelial Carcinoma
BRCA	Breast invasive carcinoma
COAD& READ	Colon adenocarcinoma & Rectum adenocarcinoma
GBM	Glioblastoma multiforme
HNSC	Head and Neck squamous cell carcinoma
KICH	Kidney Chromophobe
KIRC	Kidney renal clear cell carcinoma
KIRP	Kidney renal papillary cell carcinoma
LAML	Acute Myeloid Leukemia
LGG	Brain Lower Grade Glioma
LUAD	Lung adenocarcinoma
LUSC	Lung squamous cell carcinoma
OV	Ovarian serous cystadenocarcinoma

PRAD	Prostate adenocarcinoma
SKCM	Skin Cutaneous Melanoma
STAD	Stomach adenocarcinoma
THCA	Thyroid carcinoma
UCEC	Uterine Corpus Endometrial Carcinoma
